# Maternal high-fat diet regulates glucose metabolism and pancreatic β cell phenotype in mouse offspring at weaning

**DOI:** 10.7717/peerj.9407

**Published:** 2020-06-22

**Authors:** Jia Zheng, Ling Zhang, Ziwei Wang, Junqing Zhang

**Affiliations:** Department of Endocrinology, Peking University First Hospital, Beijing, China

**Keywords:** Maternal high-fat diet, Glucose metabolism, β cell, Offspring, Weaning

## Abstract

**Background:**

Maternal malnutrition is a critical factor in determining the risk of obesity and glucose intolerance in offspring. However, little is known about the effects of a maternal high-fat diet (HFD) on the β cell phenotype in offspring, which is a major factor in glucose homeostasis, especially during the early life of offspring.

**Methods:**

Dams were randomly fed a HFD (60% kcal from fat) or a chow diet before pregnancy and during gestation and lactation. Glucose metabolism and the β cell phenotype were assessed in male offspring at weaning.

**Results:**

Dams fed a HFD showed impaired glucose tolerance. A HFD predisposed the offspring to increased impairment of metabolic health, including obesity, glucose intolerance and insulin resistance, compared with offspring from chow diet-fed dams. Furthermore, increased islet sizes and islet densities were observed in male offspring from HFD-fed dams at weaning. There were increases in the insulin-positive area, β cell mass and β cell proliferation in male offspring from HFD-fed dams at weaning age. Next, we further determined whether a maternal HFD could affect β cell apoptosis in mouse offspring and found that there was no significant change in β cell apoptosis between the HFD and control groups.

**Conclusion:**

Our study is novel in showing that a maternal HFD predisposes offspring to impaired glucose metabolism and has a profound effect on β cell mass and proliferation in offspring mice, which is observed in mice as early as at weaning age. However, further study to clarify the underlying mechanisms is warranted.

## Introduction

The prevalence of obesity and diabetes mellitus is increasing dramatically. It is estimated that the number of people with diabetes worldwide will have increased by 45% (from 425 million to 629 million) by 2,045 (International Diabetes Federation, 2019). Of particular concern, an estimated 16.2% of pregnant women are affected by hyperglycemia during pregnancy (International Diabetes Federation, 2019). Type 2 diabetes is a complex disease involving environmental factors and genetic susceptibility. In recent years, increasing evidence has demonstrated that prenatal and early postnatal nutrition play a critical role in determining susceptibility to obesity and diabetes in offspring (Patel, Pasupathy & Poston, 2015; Pinhas-Hamiel & Zeitler, 2005; Rando & Simmons, 2015). The increase in obesity and diabetes in individuals of reproductive age has represented an enormous burden at the individual, public health, and economic levels that can propagate risks of poor metabolic health to the next generation.

Maternal nutrition during early life (prenatal and early postnatal period) can affect the risks of developing metabolic diseases during adulthood (Curhan et al., 1996; Gniuli et al., 2008; Slatkin, 2009). It is well established that a maternal high-fat diet (HFD) can contribute to the development of metabolic disturbances in offspring (Pentinat et al., 2010; Symonds et al., 2009; Williams et al., 2014). Our previous studies showed that offspring from HFD-fed dams had obesity, glucose intolerance and insulin resistance at 32 weeks of age (Zheng et al., 2016; Zheng et al., 2015). These detrimental effects have been observed in young adult and adult offspring (Bruce et al., 2009; McKay et al., 2014; Miotto et al., 2013). However, metabolic health in the early life of offspring, such as at weaning age, has not been well documented. In addition, the mechanisms underlying the detrimental interactions between early life HFD feeding and metabolic diseases in adulthood are incompletely understood.

It is well known that most organs appear to be imprinted by these early disturbances of fetal programming, including the liver (Ramaiyan & Talahalli, 2018; Zheng et al., 2014), adipose tissue (Fan et al., 2018; Ferland-McCollough et al., 2012; Loria et al., 2018), skeletal muscle (Khamoui et al., 2018; Mikovic & Lamon, 2018), central nervous system (Gomes et al., 2018; Zheng et al., 2015), and gut microbiota (Codagnone et al., 2018; Zheng et al., 2016). However, the effects on the pancreatic β cell phenotype of offspring are largely unknown, given the critical role of insulin secretion by β cells. In our study, we aimed to use a mouse model to determine the transgenerational effects of maternal HFD feeding on metabolic health and β cell phenotype in offspring as early as the weaning stage.

## Materials & Methods

### Study approval

All experimental procedures were performed in accordance with the recommendations found in the Guide for the Care and Use of Laboratory Animals and were approved by the Institutional Animal Care and Use Committee of the Peking University First Hospital (No. J201827, Beijing, China).

### Animals and diets

Five-week-old C57BL/6 female mice (*n* = 20) were purchased from Vital River Laboratory Animal Technology Co., Ltd. (Beijing, China). The male mice were obtained from the same facility as the female mice. All mice were maintained under controlled conditions (22 ± 2 °C; 12-h light/dark cycle) and acclimatized for one week. Then, the female mice were randomly assigned to a HFD or chow diet group for three weeks before breeding. The HFD derived 60% kcal from fat, 20% kcal from carbohydrate, and 20% kcal from protein, and the energy density was 5.24 kcal/g (D12492, Research Diets, New Brunswick). Female mice were bred with male mice. To control for potential differences in the sires, breeding was performed in harems (male: female = 1:2). Pregnancy was confirmed by the presence of a vaginal plug in the morning, which was considered to represent day 0.5 of pregnancy. After breeding, females were individually housed, and their assigned diet was continued throughout gestation and lactation. At postnatal day 1, all litters were culled to 6 pups to control for litter size effects on metabolic parameters.

One male offspring in each group was randomly selected from each litter and used for experimental studies (*n* = 6–8 litters in each group). At three weeks of age, mice were anesthetized by an i.p. injection of sodium pentobarbital (50 mg/kg). Blood samples were collected by piercing the right auricle. The mice were then euthanized, and their pancreas was dissected. Any surviving animals were euthanized by cervical dislocation. The female offspring were not examined in our study to prevent the influence of confounding factors related to their hormone profile and estrous cycle (Kleinert et al., 2018). In addition, our previous study showed evidence of programming effects that occurred in a sexually dimorphic manner, which was not the focus of our study (Zheng et al., 2014). Body weight was monitored weekly from birth to weaning, and food consumption by dams was measured weekly and corrected for spillage.

### Glucose tolerance test

Intraperitoneal glucose tolerance tests (IPGTTs) were performed as described previously (Zheng et al., 2016). IPGTTs were performed in dams when the offspring mice were weaned. Mice were fasted for 12 h, and the baseline blood glucose (time 0) was measured in tail vein blood samples using a glucometer and glucose test strips (Contour TS, Bayer, Beijing, China). Then, mice received an intraperitoneal injection of glucose (2 mg dextrose/g body weight), and blood glucose was measured at 15, 30, 60 and 120 min following injection. The blood glucose response to the IPGTT was calculated as the area under the curve (AUC) for each mouse.

### Insulin tolerance test

An intraperitoneal insulin tolerance test (ITT) was performed in animals fasted for 4 h. ITTs were performed in dams when the offspring mice were weaned. The baseline blood glucose (time 0) was measured as described above, and animals received an intraperitoneal injection of 1 U/kg body weight insulin (Humulin R, Lilly, USA). Subsequently, blood glucose levels were measured at 5, 10, 15, 30, 45 and 60 min following injection. The blood glucose response to the ITT was calculated as the AUC for each mouse.

### Biochemical analyses

At 3 weeks of age, mice were intraperitoneally injected with BrdU (100 mg/kg body weight) 5 h before the animals were euthanized. After centrifugation at 3,000 rpm at 4 °C for 15 min, the serum samples were separated immediately and stored at −80 °C until further analysis. Serum insulin concentrations were measured using the Mouse Ultrasensitive Insulin ELISA kit (80-INSMSU-E01, ALPCO Diagnostics, Salem, NH, USA). All samples were measured in duplicate.

### Histological analysis

The pancreas tissues were rapidly dissected, weighed, and fixed in Z-fix (Anatech Ltd. MI, USA) overnight, followed by immersion in phosphate-buffered saline (PBS). The tissue samples were dehydrated in ethanol, embedded in paraffin wax, and subsequently fixed, and serial (5 µm) paraffin-embedded tissue sections were mounted on slides for staining. The slides were stained with hematoxylin and eosin (HE). The islet size was calculated as the average of the longest and shortest diameters of each islet by ImageJ software (ImageJ, CA, USA).

### Immunofluorescence staining

Adjacent slides were deparaffinized with xylene, hydrated with an ethanol gradient and rinsed with PBS. Antigen was retrieved in citric acid buffer (pH 6.0). Sections were blocked with 5% donkey serum (Sigma-Aldrich, St Louis, MO, USA) for 1 h at room temperature. Sections were incubated overnight (4 °C) with anti-insulin (1:400; Abcam, MA, USA) and anti-Ki67 (1:50; BD Pharmingen™, USA) or anti-BrdU (1:50; Dako, Glostrup, Denmark). Sections were then incubated with Alexa Fluor 594-conjugated anti-guinea pig (1:400; Jackson ImmunoResearch Laboratories, Inc.) and Alexa Fluor 488-conjugated anti-mouse (1:400; Jackson ImmunoResearch Laboratories, Inc.) fluorescent secondary antibodies for 1 h at room temperature. Nuclei were stained with 4′,6 diamino-2-phenylindole (DAPI: 1:1000, Sigma-Aldrich, St Louis, MO, USA) for 5 min at room temperature. Finally, the tissues were mounted with fluorescent mounting medium (Dako, Glostrup, Denmark). β cell apoptosis was detected by TUNEL assays using the ApopTag^®^ Fluorescein In Situ Apoptosis Detection Kit (Millipore, MA, USA) according to the manufacturer’s protocol. Cell counting was manually performed in a blinded fashion. BrdU (+) or Ki67 (+) β cells were assessed by immunofluorescence microscopy. Insulin (+) cells showing nuclear DAPI staining were considered β cells. Insulin (+) cells showing colocalized nuclear staining for DAPI (+) and Ki67 (+) or BrdU (+) were considered proliferating β cells. Insulin (+) cells showing colocalized nuclear staining for DAPI (+) and TUNEL (+) were considered apoptotic β cells. The double-positive staining of insulin (+)/BrdU ( +), insulin (+)/Ki67 (+) and insulin (+)/TUNEL (+) were confirmed in randomly selected cells in all experiments.

### Quantification of β cell mass, proliferation and apoptosis

Photographed sections were analyzed with imaging software (ImageJ, CA, USA) to measure insulin-positive areas and to determine the number of nuclei in the insulin-positive areas. The β cell mass (mg) was calculated by multiplying the relative insulin (+) area (the percentage of the insulin-positive area over the total pancreas area) by the pancreas weight (mg). The total β cell, Ki-67 (+) β cell, BrdU (+) β cell and TUNEL (+) β cell numbers were determined from the β cell counts in each section after correction for cut nuclei. β cell proliferation was calculated as the percentage (%) of Ki-67 (+) and BrdU (+) β cells over the total number of β cells per section. β cell apoptosis was calculated as the percentage (%) of TUNEL (+) β cells over the total number of β cells per section.

### Statistical analysis

Data are displayed as the mean ± standard error of the mean (SEM). GraphPad Prism software (La Jolla, CA) was utilized to analyze significant differences using Student’s *t*-test or analysis of variance (ANOVA), where appropriate. Statistical significance was defined as *P* < 0.05.

## Results

### Effects of HFD on the metabolic profile in dams

**Figure 1:**
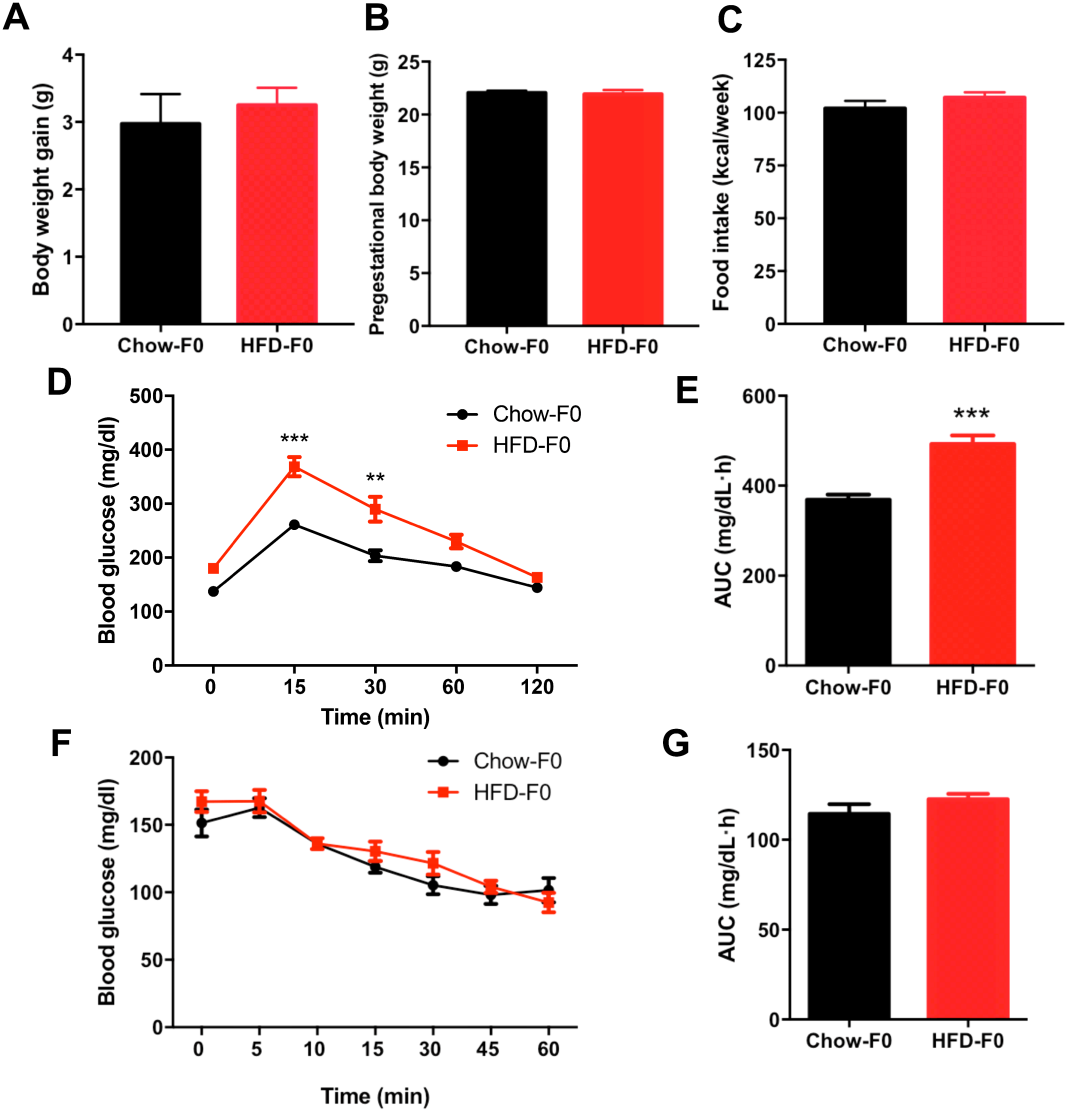
Effects of diets on metabolic profile in dams. (A) Body weight gain during 3 weeks before pregnancy; (B) Pre-gestational body weight; (C) Food intake; (D) Intraperitoneal glucose tolerance test (IPGTT) of dams; (E) AUC during the glucose tolerance test of dams; (F) Insulin tolerance test (ITT) of dams; (G) AUC during the insulin tolerance test of dams. AUC: area under the curve, HFD: high-fat diet; Dams were noted as F0; Data represented as the mean ±  SEM. **P < 0.01, ***P < .001 vs. Chow, *n* = 12 to 16, per group.

There was no difference in body weight gain or pregestational body weight before breeding between HFD- and chow diet-fed dams (Figs. 1A–1B). There was no significant difference in food intake between dams fed a HFD and those fed a chow diet (Fig. 1C). To eliminate the stress of pregnancy and delivery, we did not perform glucose tolerance tests during pregnancy. IPGTT and ITT were performed in dams when offspring mice were weaned, and they revealed impaired glucose tolerance in HFD dams, with an increased AUC (Figs. 1D–1E). However, blood glucose levels indicating insulin tolerance were indistinguishable between groups (Figs. 1F–1G).

### Impaired glucose metabolism in male offspring from HFD-fed dams at weaning

**Figure 2:**
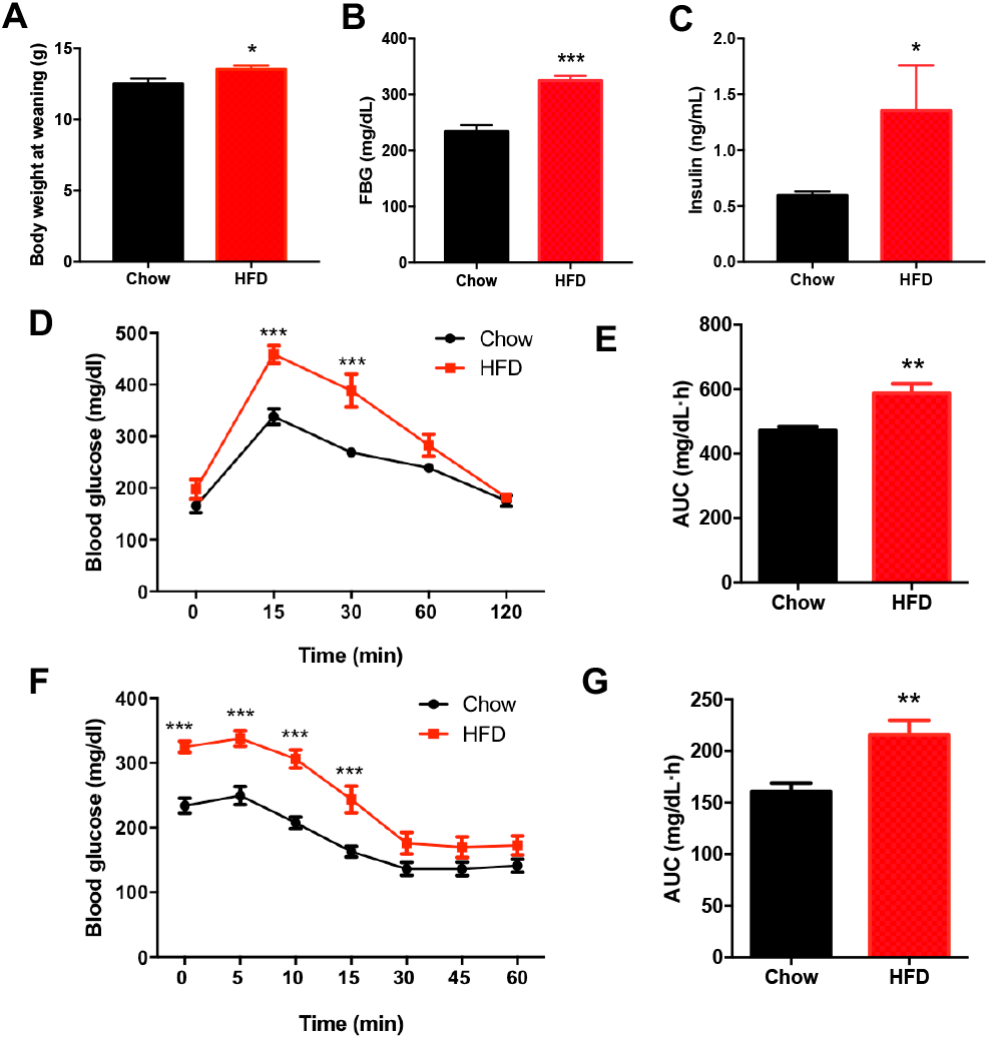
Impaired glucose metabolism in male offspring from HFD-fed dams at weaning. (A) Body weight at weaning; (B) Fasting blood glucose (FBG); (C) Fasting insulin concentration; (D) Intraperitoneal glucose tolerance test (IPGTT) of male offspring at weaning; (E) AUC during the glucose tolerance test; (F) Insulin tolerance test (ITT) of male offspring at weaning; (G) AUC during the insulin tolerance test of offspring. AUC: area under the curve, HFD: high-fat diet; Data represented as the mean ± SEM. *P < 0.05, **P < 0.01, ***P < .001 vs. Chow, *n* = 6 − 8 litters in each group, one male offspring per litter.

There was no significant difference in the birth weight of offspring between dams fed a HFD and those fed a chow diet. At weaning, male offspring of HFD-fed dams exhibited significantly higher body weight compared with the offspring of dams fed a chow diet (*P* < 0.05, Fig. 2A). Fasting blood glucose (FBG) (*P* < 0.001, Figs. 2B–2C) and insulin concentration (*P* < 0.05, Figs. 2B–2C) were significantly increased in male offspring of HFD-fed dams at weaning. Consistently, the blood glucose levels of the male offspring in the HFD group were increased at 15 min and 30 min after intraperitoneal glucose administration (*P* < 0.001, Fig. 2D), and these male offspring also had an increased AUC (*P* < 0.01, Fig. 2E). Furthermore, the insulin tolerance test showed higher blood glucose levels at 0 min, 5 min, 10 min and 15 min in male offspring of HFD-fed dams compared with those in male offspring in the chow group (*P* < 0.001, Fig. 2F), indicating decreased insulin sensitivity (*P* < 0.01, Fig. 2G).

### Increased islet size and density in male offspring from HFD-fed dams

**Figure 3:**
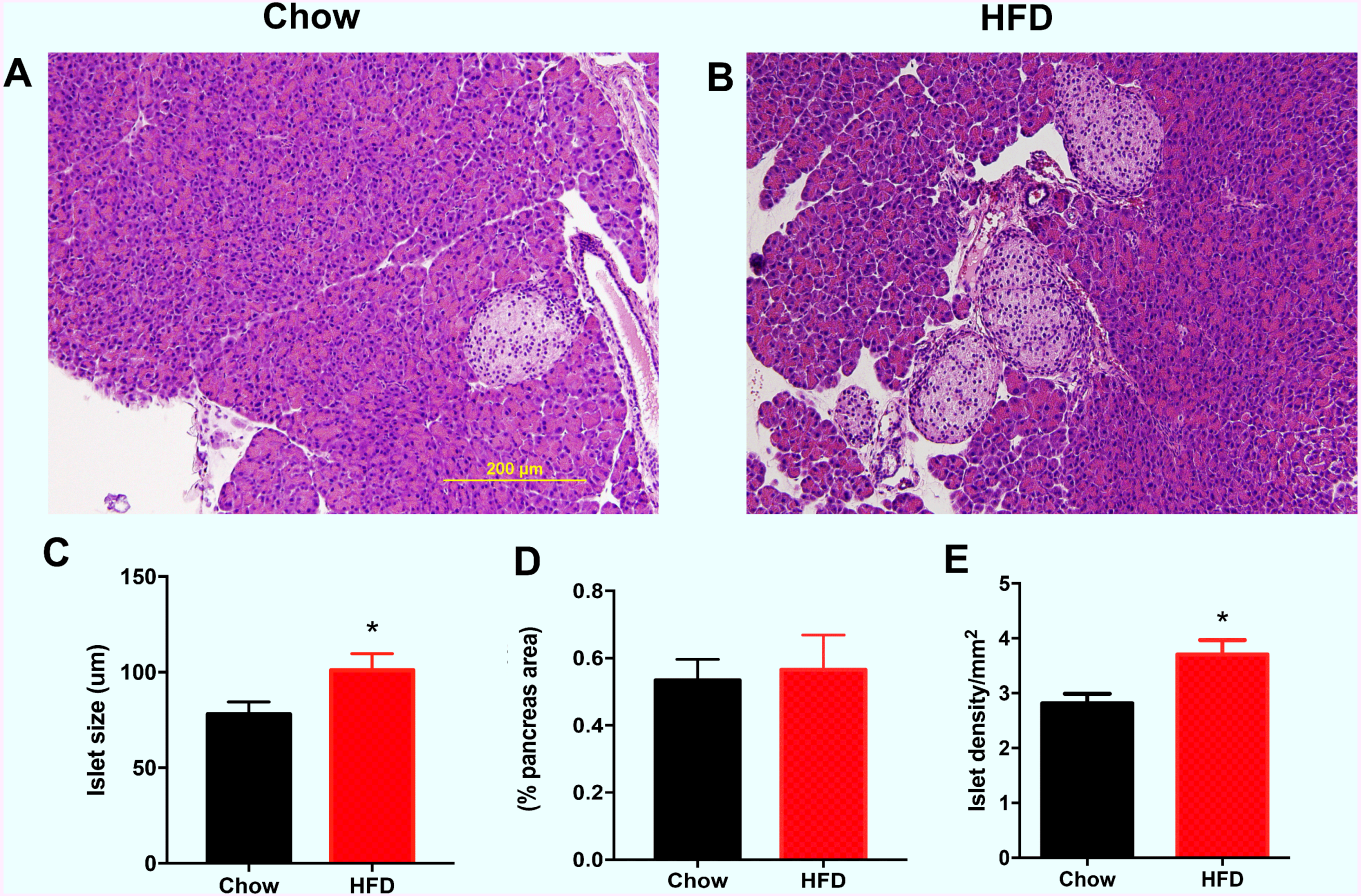
Effect of maternal over-nutrition on islet morphology in male offspring at weaning. (A–B) Representative images of HE staining in pancreatic cross sections. Scale: 200 um. (C) Quantitative analysis of islets size. The islet size was calculated by the average of the longest and shortest diameters of each islet. (D) Total islet area (% pancreas areas). (E) Islet density (calculated by total number of islets/pancreas areas (mm2)). HFD: high-fat diet. Data represented as the mean ± SEM. *P < 0.05 vs. Chow, *n* = 6 − 8 litters in each group, one male offspring per litter.

As shown in Fig. 3A, the representative images indicate that the male offspring from HFD-fed dams had larger islets according HE staining than the male offspring in the chow group. Increased islet sizes were found in male offspring from HFD-fed dams at weaning (*P* < 0.05, Fig. 3B). There was no difference in the total islet area (% pancreas areas) in male offspring at weaning between the two groups (Fig. 3C). However, we found that islet density was significantly increased in male offspring from HFD-fed dams at weaning age compared with that in male offspring in the control group (*P* < 0.05, Fig. 3D).

### Increased β cell mass in male offspring from HFD-fed dams

**Figure 4:**
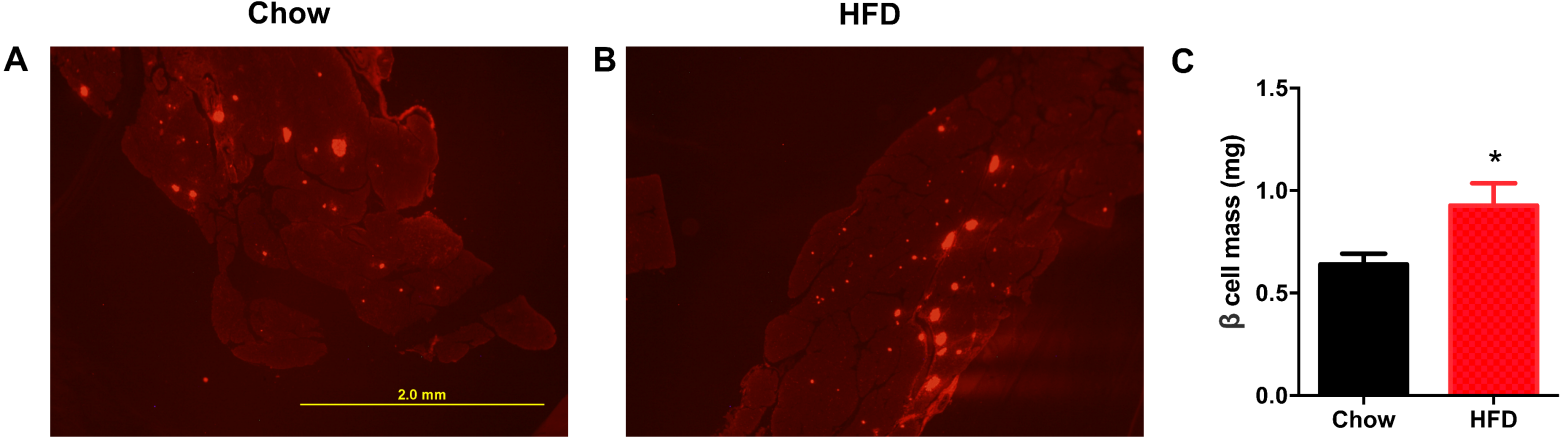
Higher β cell mass in male offspring from HFD-fed dams at weaning. (A–B) Representative images of insulin-positive areas in pancreatic cross sections immunostained with anti-insulin antibody. (C) β cell mass quantification. HFD: high-fat diet. Data represented as the mean ± SEM. *P < 0.05 vs. Chow, *n* = 6 − 8 litters in each group, one male offspring per litter.

Next, we aimed to determine whether a maternal HFD could regulate the β cell mass in offspring at weaning. As shown in Fig. 4A, the red area was insulin-positive and was increased in male offspring from HFD-fed dams. We found that the β cell mass was significantly increased in offspring from HFD-fed dams (*P* < 0.05, Fig. 4B).

### Effect of a maternal HFD on β cell proliferation and apoptosis in male offspring at weaning age

**Figure 5:**
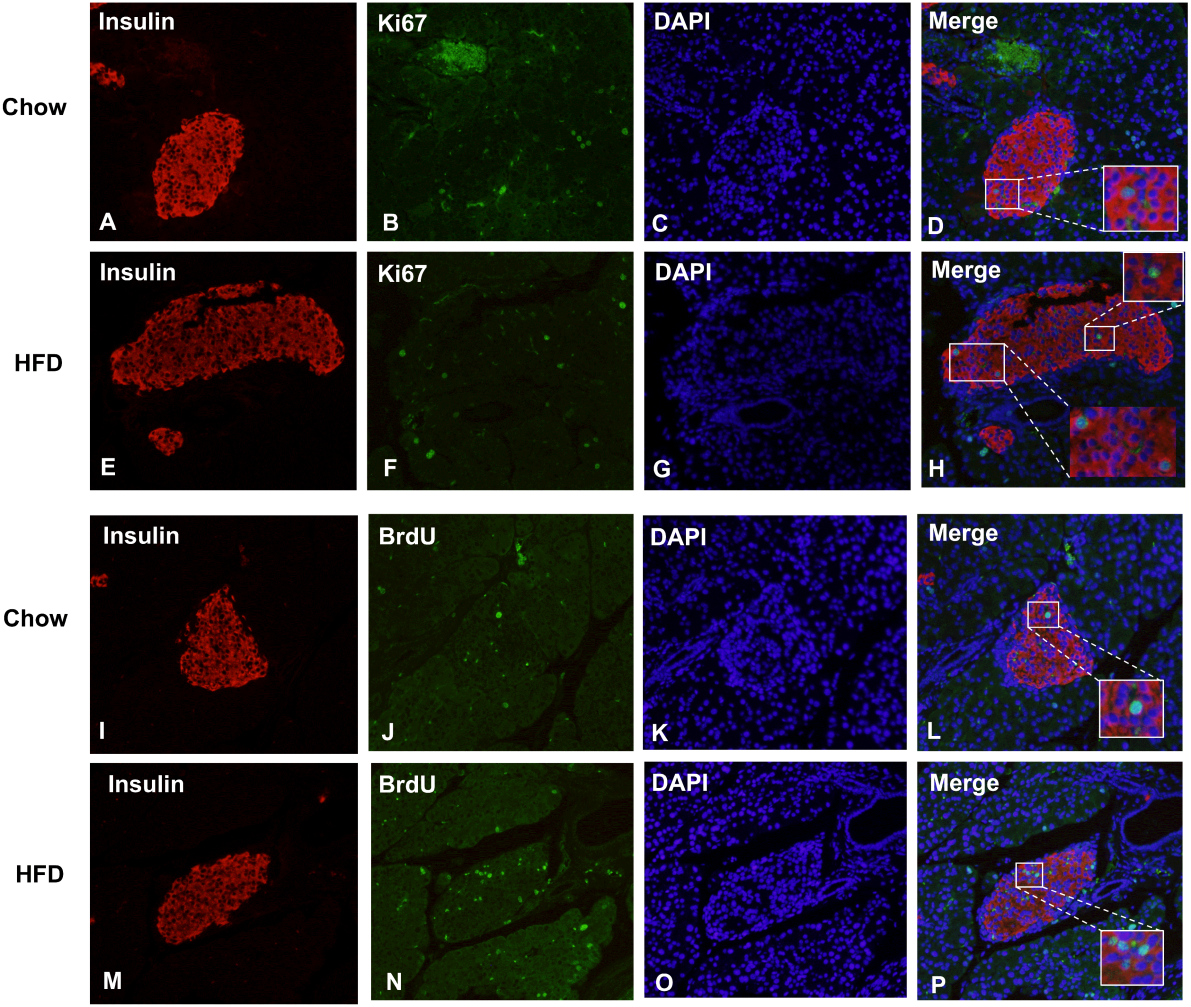
Maternal HFD predisposes higher β cell proliferation in male offspring at weaning. (A–H) Representative images of proliferating cells in pancreatic sections immunostained with insulin/Ki67/DAPI in Chow and HFD groups. (I–P) Representative images of proliferating cells in pancreatic sections immunostained with insulin/BrdU/DAPI in Chow and HFD groups. HFD: high-fat diet. Feel free to contact me if you have any questions

**Figure 6:**
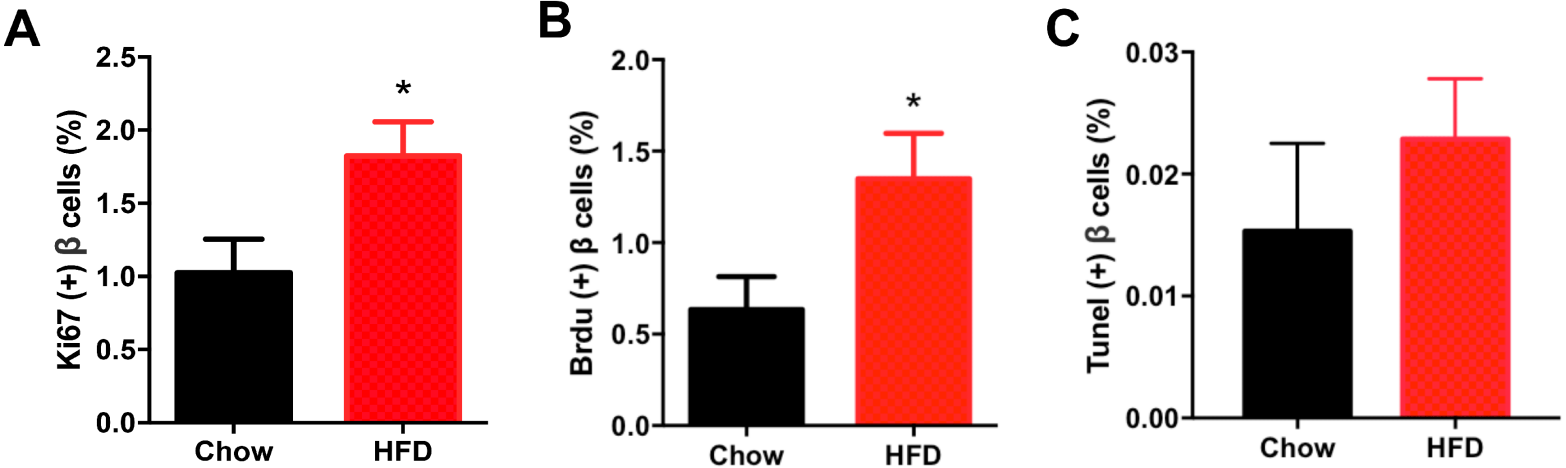
Effect of maternal over-nutrition on β cell proliferation and apoptosis in offspring at weaning age. (A) Quantification of Ki67(+) insulin(+) cells: at least 5,000 insulin(+) cells were counted for each mouse. (B) Quantification of BrdU(+) insulin(+) cells: at least 5,000 insulin(+) cells were counted for each mouse. (C) Quantification of TUNEL(+) insulin(+) cells: at least 5,000 insulin(+) cells were counted for each mouse. HFD: high-fat diet. Data represent mean ± SEM. ^∗^*P* < 0.05 vs. Chow, *n* = 6–8 litters in each group, one male offspring per litter.

Figure 5 shows representative images of proliferating cells in pancreatic sections immunostained with insulin/Ki67/DAPI and insulin/BrdU/DAPI as denoted. St least 5,000 insulin (+) cells were counted for each mouse for the calculation. The immunofluorescence costaining of Ki67 and insulin showed increased β cell proliferation in male offspring from HFD-fed dams than that in chow-fed dam offspring (*P* < 0.05, Fig. 6A). To confirm the difference in β cell proliferation, we used another proliferation marker, BrdU, to determine the β cell proliferation rate. BrdU can be incorporated into the newly synthesized DNA of replicating cells (during the S phase of the cell cycle, in which DNA is replicated), thus indicating cells that are actively replicating their DNA. Consistent with the results of Ki67 staining, an increased percentage of insulin (+)/BrdU (+)/DAPI (+) β cells was observed in male offspring from HFD-fed dams (*P* < 0.05, Fig. 6B). Because β cell apoptosis can also affect the β cell mass, we further measured the β cell apoptosis rate and found no differences in the β cell apoptosis rate in the 3-week-old male offspring (*P* < 0.01, Fig. 6C).

## Discussion

In recent years, it has been well established that risk factors for obesity and type 2 diabetes can originate from alterations in growth and metabolism during critical windows of prenatal and early postnatal development. Injury to the intrauterine environment (including both overnutrition and undernutrition) have been demonstrated to affect health in offspring, which can alter responses to environmental challenges and thus increase the predisposition to metabolic diseases (Langley-Evans, 1996; Zhang et al., 2009).

The detrimental effects of maternal overnutrition and obesity on the metabolic health of offspring have been well established in human (Barker et al., 1989; Hales et al., 1991; Pinhas-Hamiel & Zeitler, 2005; Ravelli, Stein & Susser, 1976; Whitaker et al., 1997) and animal studies (Isganaitis et al., 2009; Masuyama & Hiramatsu, 2012; Morris & Chen, 2009; Stanford et al., 2015; Woo et al., 2011). However, most studies have focused on the detrimental metabolic effects in young adult, adult and even elderly offspring. Here, we demonstrate that a maternal HFD before pregnancy and during gestation and lactation predisposed offspring to the impairment of metabolic health, including obesity, glucose intolerance and insulin resistance, as early as the weaning stage. It also resulted in an increase in the islet size and islet density in offspring from HFD-fed dams. Furthermore, increased pancreatic β cell mass and β cell proliferation were both observed in male offspring from HFD-fed dams at weaning age. Thus, a maternal HFD was able to regulate glucose metabolism and the pancreatic β cell phenotype in offspring mice as early as the weaning stage.

Our study aimed to investigate the effects of a maternal HFD on β cells in offspring as early as the weaning stage, which is different from the aims of other studies. Most studies have focused on the long-term effects of a maternal HFD feeding on pancreatic β cell function in offspring. A maternal HFD induced insulin resistance and deterioration of pancreatic β-cell function in adult offspring at 20 weeks of years, accompanied by decreased insulin content and pancreatic homeobox 1 (PDX-1) mRNA levels in isolated islets. Bringhenti et al. showed that feeding dams a HFD was responsible for remodeling the islet structure in 6-month-old offspring mice via the migration of some *α* cells from the edge to the core of the islet in association with an increase in the masses of *α* cells, β cells, and islets (Bringhenti et al., 2016). In another study, the offspring mice of HFD-fed dams were evaluated at 50 weeks of age and were characterized by significantly reduced basal and glucose stimulated insulin secretion from islets (Han et al., 2005). Thus, a maternal HFD induces a change in the developmental programming of the islet with permanent consequences for β cell function.

The mechanisms by which maternal malnutrition impairs islet function in offspring are unknown. Pancreatic β cells can be described as simple cells that perform two functions: continuous sensing of blood glucose levels and appropriate secretion of insulin (Granot et al., 2009). Regulation of the β cell mass is dynamic and tightly controlled to meet the demand for insulin. The homeostatic control of the β cell mass in both normal and pathophysiological conditions is based on the balance of cell proliferation, cell growth, and cell death (Finegood, Scaglia & Bonner-Weir, 1995). Our study found that the β cell mass was significantly increased in offspring from HFD-fed dams and was accompanied by increased β cell proliferation. This can be explained by the fact that hyperglycemia in the HFD group could signal to β cells that more insulin was needed, so compensation could occur, including functional as well as enhanced β cell proliferation (Bonner-Weir, 2000). In addition to the pregnancy period, the lactation period is also very important in glucose metabolism in offspring. Breast milk, as the main form of neonatal nutrition, has been shown to reduce the risk of developing diabetes for human infants (Bartok & Ventura, 2009). High-fat diet-associated metabolites are directly transferred to offspring via alterations in the quality or quantity of milk production during lactation (Saben et al., 2014). Bautista et al. showed that maternal obesity induced by an obesogenic diet negatively affects maternal liver and mammary gland function and causes significant changes in milk composition (Bautista et al., 2016). Thus, we speculate that breast milk consumption during the lactation period may affect the development and maturation of the endocrine pancreas.

To our knowledge, this is the first report to show that a maternal HFD has a profound effect on islet morphology and pancreatic β cell mass in offspring, which may explain the impaired glucose tolerance and insulin sensitivity of offspring observed as early as at the weaning age. However, potential limitations should be considered in our study. First, our data regarding the β cell phenotype are preliminary and descriptive. Further studies should be performed to demonstrate the mechanisms that regulate β cell mass and proliferation mainly by focusing on β cell proliferation-related proteins and the insulin signaling pathway. Second, our present study mainly focused on the detrimental effects of a maternal HFD in the early life of offspring, and there were no data on the long-term effects in adulthood in our model. The long-term effects of metabolic abnormalities and pancreatic β cell function must be further examined. Third, it is still unknown whether the critical time window for a maternal HFD occurs in the pre-gestation period, gestation period, or lactation period. Our current work aims to determine the specific time window of the effects of a maternal HFD on glucose metabolism in offspring.

## Conclusions

In conclusion, our study is novel in showing that a maternal HFD has a profound effect on metabolic health and the pancreatic β cell phenotype, especially the β cell mass and β cell proliferation, in offspring as early as the weaning stage. However, further studies should be performed to demonstrate the mechanisms that regulate β cell mass and proliferation. A better understanding of the role and action of β cell function can help assess the contribution to the transgenerational effects of a maternal HFD. This is expected to facilitate the development of a target for the prevention and intervention of metabolic diseases during the early stage of life.

##  Supplemental Information

10.7717/peerj.9407/supp-1 Supplemental Information 1 Raw data of metabolic phenotype in offspring

## Additional Information and Declarations

### Data Availability

The following information was supplied regarding data availability: The raw measurements are available in the Supplementary Files.
